# Comparative West Nile virus detection in organs of naturally infected American Crows (Corvus brachyrhynchos).

**DOI:** 10.3201/eid0704.010430

**Published:** 2001

**Authors:** N A Panella, A J Kerst, R S Lanciotti, P Bryant, B Wolf, N Komar

**Affiliations:** Centers for Disease Control and Prevention, Fort Collins, CO 80522, USA. nap4@cdc.gov

## Abstract

Widespread deaths of American Crows (Corvus brachyrhynchos)were associated with the 1999 outbreak of West Nile (WN) virus in the New York City region. We compared six organs from 20 crow carcasses as targets for WN virus detection. Half the carcasses had at least one positive test result for WN virus infection. The brain was the most sensitive test organ; it was the only positive organ for three of the positive crows. The sensitivity of crow organs as targets for WN virus detection makes crow death useful for WN virus surveillance.


### Comparative West Nile Virus Detection in Organs of Naturally Infected American Crows (*Corvus brachyrhynchos*)

**Nicholas A. Panella,* Amy J. Kerst,* Robert S. Lanciotti,* Patricia Bryant,† Bruce Wolf,† and Nicholas Komar***
*Centers for Disease Control and Prevention, Fort Collins, Colorado, USA; and †New Jersey Department of Health and Senior Services Virology Program, Trenton, New Jersey, USA

Widespread deaths of American Crows (*Corvus brachyrhynchos*) were associated with the 1999 outbreak of West Nile (WN) virus in the New York City region. We compared six organs from 20 crow carcasses as targets for WN virus detection. Half the carcasses had at least one positive test result for WN virus infection. The brain was the most sensitive target organ; it was the only positive organ for three of the positive crows. The sensitivity of crow organs as targets for WN virus detection makes crow death useful for WN virus surveillance.

The 1999 outbreak of West Nile (WN) virus in the New York City area (1) was associated with the deaths of thousands of American Crows (*Corvus brachyrhynchos*), which appeared to be highly susceptible to the virus. Local health authorities selected some of these dead birds for laboratory testing. Generally, brain tissue was targeted for virus isolation as a method of surveillance (2). Although WN virus has frequently been isolated from brain tissue, a rigorous comparison of the brain to other organs of the American Crow has not been undertaken. Accordingly, we compared the sensitivity of the brain with that of other crow organs as targets for WN virus detection by both virus isolation and RNA detection.

### The Study

From 20 crow carcasses collected in New Jersey during September and October 1999, we removed sections of brain, liver, spleen, kidney, heart, and lung for WN virus detection by plaque assay and TaqMan reverse transcription-polymerase chain reaction (RT-PCR) (3). The samples were prepared by macerating approximately 0.5 $cm^3$ of tissue in 1.8 mL of BA-1 (composed of M-199 Hanks salts, 29.2 mg/mL L-glutamine, 0.05 M Tris-HCl, pH 7.6, 1% bovine serum albumin, 350 mg/L sodium bicarbonate, 100 units/mL penicillin, 100 mg/L streptomycin, and 100 µg/mL Fungizone) diluent in a glass Ten Broeck tissue grinder (Bellco Glass, Inc., Vineland, NJ). Virus isolation was attempted in duplicate 100-µL aliquots by Vero cell plaque assay. A 5-µL aliquot from each sample was tested by TaqMan RT-PCR assay, which quantitates WN virus RNA. Sensitivity of each assay for detecting WN virus or RNA in each organ was determined by using only the WN virus-infected carcasses as denominator in the calculations.

Address for correspondence: Nick Panella, Centers for Disease Control and Prevention, P.O. Box 2087, Fort Collins, CO 80522, USA; fax: 970-221-6476; e-mail: nap4@cdc.gov

One hundred nineteen tissue samples from 20 crows were assayed for WN virus (Table). Positive test results for WN virus infection were obtained for 10 of the 20 carcasses. WN virus was most often isolated from brain (8 [80%] of 10) and heart (6 [67%] of 9), while WN virus RNA was most frequently detected in brain (10 [100%] of 10) and liver and kidney (each 8 [80%] of 10). The TaqMan assay identified WN virus RNA in seven tissue samples that tested negative by plaque assay, including two brain tissue samples of crows from which all other organ tissues had tested negative. Tissues from the three crows for which only brain provided positive RNA detection were confirmed positive by repeat-testing in triplicate with three different TaqMan RT-PCR primer pairs. WN virus was then isolated by plaque assay from approximately 1 g of brain tissue from one of these specimens (NJN-37, data not shown).

### Conclusions

The findings suggest that the brain is the most sensitive target organ (of those tested) from crow carcasses for detecting WN virus with both detection assays ($p = 0.0816$). However, heart, lung, liver, kidney, and spleen were all good sources of WN virus with both assays. (The liver was not a good source of detection with the plaque assay.) Using the TaqMan assay, we were able to identify WN virus RNA in several tissue specimens that were negative by Vero plaque assay. The Taqman assay may be especially useful when organs from necropsied crows no longer contain live virus.

If WN virus continues to spread, rapid detection will be an important public health issue. Since WN virus attacks various internal organs in birds (4), viscera from dead crows can be used to detect the virus in a surveillance program. Our findings, consistent with those of earlier studies, indicate that the brain is the most frequently affected organ among WN virus-infected birds (4) and support the continued use of the brain as the organ of choice from dead crows for surveillance and as a target for WN virus detection in diagnostic assays.

Table. Amount of virus detected by Vero plaque assay and TaqMan reverse transcriptase polymerase chain reaction in American Crow organs

| Crow number | Heart | Kidney | Liver | Lung | Spleen | Brain |
|---|---|---|---|---|---|---|
| NJN 5 | +++[a]/3.40E+03[b] | +++/5.90E+04 | ++ /8.48E+04 | +++/2.42E+04 | +++/4.09E+03 | +++/5.41E+03 |
| NJN 6 | ++/2.36E+03 | ++/1.12E+04 | -[c]/1.10E+02 | -/4.61E+02 | +/2.02E+02 | ++/3.12E+02 |
| NJN 7 | ++/9.57E+03 | ++/5.52E+03 | -/7.61E+01 | +/3.46E+03 | +/1.26E+02 | ++/1.76E+03 |
| NJN 8 | +++/2.31E+05 | +++/5.41E+04 | +++/5.61E+05 | +++/3.20E+04 | +++/4.08E+04 | +++/6.18E+04 |
| NJN 9 | +++/2.94E+04 | +++/1.96E+05 | +/2.15E+05 | +++/4.36E+05 | +++/1.17E+05 | +++/1.78E+05 |
| NJN 11 | +++/3.62E+04 | +++/1.06E+04 | +++/1.06E+03 | +++/1.31E+04 | +++/1.07E+03 | +++/2.15E+04 |
| NJN 13 | NT/NT | +/2.54E+02 | -/1.50E+00 | -/1.68E+02 | -/- | +/9.28E+01 |
| NJN 29 | -/- | -/- | -/- | -/- | -/- | ++/6.67E+00 |
| NJN 30 | -/- | -/- | -/- | -/- | -/- | -/- |
| NJN 33 | -/- | -/- | -/- | -/- | -/- | -/ 3.24E+00 |
| NJN 37 | -/- | -/- | -/- | -/- | -/- | -/ 2.10E-01 |
| NJN 40 | -/- | -/- | -/- | -/- | -/- | -/- |
| NJN 41 | -/- | -/- | -/- | -/- | -/- | -/- |
| NJN 43 | -/- | -/- | -/- | -/- | -/- | -/- |
| NJN 44 | -/- | -/- | -/- | -/- | -/- | -/- |
| NJN 51 | -/- | -/- | -/- | -/- | -/- | -/- |
| NJN 57 | -/- | -/- | -/- | -/- | -/- | -/- |
| NJN 62 | -/- | -/- | -/- | -/- | -/- | -/- |
| NJN 75 | -/- | -/- | -/- | -/- | -/- | -/- |
| NJN 95 | -/- | -/- | -/- | -/- | -/- | -/- |

[a]Vero cell plaque assay: +++ = ≥100 PFU/200 µL, ++ = 10–100 PFU/200 µL, + = ≤10 PFU/200 µL.

[b]TaqMan RT-PCR assay: PFU equivalents/5 µL.

[c]Negative.

NT = Not tested.

Mr. Panella is a biologist in the Arbovirus Diseases Branch, Division of Vector-Borne Infectious Diseases, Centers for Disease Control and Prevention, Fort Collins, CO, USA. He has participated in West Nile virus outbreak investigations and has contributed to studies of West Nile virus ecology in the United States.

### References

1. Komar N. West Nile viral encephalitis. Rev Sci Tech 2000;19:166-76.
2. Eidson M, Komar N, Sorhage F, Nelson R, Talbot T, Mostashari F, et al. Crow deaths as a sentinel surveillance system for West Nile virus in the northeastern United States, 1999. Emerg Infect Dis 2001;7:615-20.
3. Lanciotti RS, Kerst AJ, Nasci RS, Godsey MS, Mitchell CJ, Savage HM, et al. Rapid detection of West Nile virus from human clinical specimens, field-collected mosquitoes, and avian samples by a Taqman reverse transcriptase PCR assay. J Clin Microbiol 2000;38:4066-71.
4. Steele KE, Linn MJ, Schoepp RJ, Komar N, Geisbert TW, Manduca RM, et al. Pathology of a fatal West Nile virus infections in native and exotic birds during the 1999 outbreak in New York City. J Vet Pathol 2000;37:208-24.
